# Statistical Optimization of Laccase Production and Delignification of Sugarcane Bagasse by *Pleurotus ostreatus* in Solid-State Fermentation

**DOI:** 10.1155/2015/181204

**Published:** 2015-06-09

**Authors:** Susan Grace Karp, Vincenza Faraco, Antonella Amore, Luiz Alberto Junior Letti, Vanete Thomaz Soccol, Carlos Ricardo Soccol

**Affiliations:** ^1^Department of Bioprocess Engineering and Biotechnology, Federal University of Paraná, Coronel Francisco H. dos Santos Avenue, 210, 81531-990 Curitiba, PR, Brazil; ^2^Biotechnology Program, Positivo University, Pedro Viriato Parigot de Souza Street, 5300, 81280-330 Curitiba, PR, Brazil; ^3^Department of Chemical Sciences, University of Naples “Federico II”, Monte S. Angelo University Complex, Via Cintia 4, 80126 Naples, Italy

## Abstract

Laccases are oxidative enzymes related to the degradation of phenolic compounds, including lignin units, with concomitant reduction of oxygen to water. Delignification is a necessary pretreatment step in the process of converting plant biomass into fermentable sugars. The objective of this work was to optimize the production of laccases and to evaluate the delignification of sugarcane bagasse by *Pleurotus ostreatus* in solid-state fermentation. Among eight variables (pH, water activity, temperature, and concentrations of CuSO_4_, (NH_4_)_2_SO_4_, KH_2_PO_4_, asparagine, and yeast extract), copper sulfate and ammonium sulfate concentrations were demonstrated to significantly influence laccase production. The replacement of ammonium sulfate by yeast extract and the addition of ferulic acid as inducer provided increases of 5.7- and 2.0-fold, respectively, in laccase activity. Optimization of laccase production as a function of yeast extract, copper sulfate, and ferulic acid concentrations was performed by response surface methodology and optimal concentrations were 6.4 g/L, 172.6 *μ*M, and 1.86 mM, respectively. Experimentally, the maximum laccase activity of 151.6 U/g was produced at the 5th day of solid-state fermentation. Lignin content in sugarcane bagasse was reduced from 31.89% to 26.36% after 5 days and to 20.79% after 15 days by the biological treatment of solid-state fermentation.

## 1. Introduction

Laccases are blue multicopper oxidases able to oxidize a variety of phenolic compounds and, in the presence of a mediator (e.g., 2,2′-azinobis-3-ethylbenzthiazoline-6-sulfonate or ABTS), also nonphenolic compounds [1–3]. It is known that the production of laccases by fungi, especially the white-rot basidiomycetes, can be affected by the type and concentration of the carbon and nitrogen sources and also by the presence of copper and organic compounds that act as inducers of laccase activity [4–7].

Laccases are indicated for several applications in different sectors, that is, in the food and beverages industry to remove undesired phenolics which are responsible for browning, haze formation, and turbidity, in the pulp and paper industry, for biopulping and biobleaching processes, and in the textile industry, for dyes decolourization, and there are also applications in the fields of nanotechnology, bioremediation, and synthetic chemistry [8, 9].

The utilization of agroindustrial wastes as substrates for fermentative processes producing high added value products (i.e., enzymes, ethanol, single-cell protein, mushrooms, organic acids, amino acids, biologically active secondary metabolites) has been widely explored, since they are easily available and rich in carbon and often present disposal problems [10–12]. Sugarcane bagasse is an agroresidue generated in high amount (186 million tons/year) by the sugar and alcohol industry in Brazil. It is a porous residue of cane stalks left over after the crushing and extraction of the juice from sugarcane and is composed of 19–24% lignin, 27–32% hemicellulose, 32–44% cellulose, and 4.5–9% ashes [13]. Sugar mills generate approximately 270–280 kg of bagasse (50% moisture) per metric ton of sugarcane [14].

One of the main challenges in the utilization of lignocellulosic biomass in fermentative processes is the transformation of the complex polysaccharides into simple sugars that can be assimilated by microorganisms. This can be achieved by chemical or enzymatic hydrolysis, preceded by appropriate pretreatments that enhance the efficiency of hydrolysis by lignin removal [15].

Delignification can be performed by thermochemical processes or by the biological route, using enzymes or microorganisms. The advantages of biological delignification over the thermochemical methods may include mild reaction conditions, higher product yields, fewer side reactions, less energy demand, and less reactor resistance to pressure and corrosion [16].

Lignin decomposition in nature is primarily attributed to the metabolism of microorganisms. Among all other organisms, white-rot basidiomycetes degrade lignin more rapidly and extensively than other groups [17] through the cooperative action of several ligninolytic enzymes (laccases, manganese peroxidases, and lignin peroxidases) [18].

Some white-rot fungi such as* Ceriporiopsis subvermispora*,* Phellinus pini*,* Phlebia* spp., and* Pleurotus* spp. preferentially attack lignin more readily than hemicellulose and cellulose. Many white-rot fungi, however, such as* Trametes versicolor*,* Heterobasidion annosum,* and* Irpex lacteus,* exhibit a pattern of simultaneous decay characterized by degradation of all cell wall components [3].

Solid-state fermentation is an interesting technology to be applied in the valorization of agroindustrial residues and can be economically feasible for the production of many biotechnological products [12]. It is also an interesting process to perform biological delignification because it mimics the natural environment of lignin-degrading fungi. The advantages of the solid-state fermentation process over submerged fermentation include smaller fermenter volume, lower sterilization energy costs, easier aeration, reduced or eliminated costs for stirring and effluent treatment, higher product stability, lower catabolic repression, less favorable environment for many bacteria, and lower risk of contamination [12, 16, 19].

The objective of this work was to optimize the fermentation conditions for the production of laccases and to evaluate the delignification of sugarcane bagasse, through the process of solid-state fermentation, using a selected strain of* Pleurotus ostreatus* (coded Pl 22 Em).

## 2. Material and Methods

### 2.1. Characterization of the Sugarcane Bagasse

The sugarcane bagasse, generated by the sugar and alcohol industry, was provided by the private company Ourofino Agronegócio, located in the region of Ribeirão Preto, São Paulo, Brazil. The bagasse was previously washed with water and dried at 60°C and the portion presenting particle sizes greater than 2 mm was grinded in a knife mill. Separation of the fractions presenting different particle sizes was performed by sieving. Contents of lignin, total extractives, ashes, and moisture were determined according to the TAPPI norms T222, T264, T413, and T264, respectively. Holocellulose content was calculated by difference.

### 2.2. Solid-State Fermentations

Erlenmeyer flasks containing 1 g of sugarcane bagasse (particle size between 0.8 and 2 mm 50% and <0.8 mm 50%) were autoclaved and humidified with a sterilized saline solution presenting the following constant composition: MgSO_4_·7H_2_O (0.3 g/L), FeSO_4_·7H_2_O (0.005 g/L), MnSO_4_·H_2_O (0.00156 g/L), ZnSO_4_·7H_2_O (0.0014 g/L), CaCl_2_ (0.3 g/L), and CoCl_2_ (0.002 g/L) [20]. For the experiments designed according to Plackett-Burman (Table 1), the saline solution presented some differences regarding the following variables: CuSO_4_·5H_2_O as the inducer (0, 75 or 150 *μ*M, concentrations defined according to previous experiments), (NH_4_)_2_SO_4_ as the nitrogen source (1.5, 2.0 or 2.5 g/L), KH_2_PO_4_ as the source of potassium and phosphorus (1, 1.5 or 2 g/L), asparagine as the supplementary aminoacid (0, 0.3 or 0.6 g/L), yeast extract as the source of vitamins and aminoacids (0, 0.25 or 0.5 g/L), and pH (5.0, 5.5 or 6.0). The pH was adjusted with HCl 1 M or NaOH 1 M. These variables were chosen on the basis of the composition of the basidiomycetes synthetic medium. In order to evaluate the effect of water activity (*A*_*w*_) and temperature, the bagasse was humidified with different volumes of saline solution (10, 15, and 20 mL/g, corresponding to initial *A*_*w*_ of 0.993, 0.996, and 0.999, resp.) and the cultures were incubated at 25, 29, and 33°C. For the subsequent experiments, the concentration of KH_2_PO_4_ and the pH were set at 1.5 g/L and 5.5, respectively; no asparagine was added; initial *A*_*w*_ was fixed at 0.993 or 10 mL/g and temperature at 29°C. The studied variables were nitrogen source (yeast extract, 1.96–12.04 g/L or (NH_4_)_2_SO_4_, 2.5 g/L), inorganic inducer (CuSO_4_, 24–276 *μ*M), and organic inducer (ferulic acid, 0.32–3.68 mM, added after 48 h of fermentation).

The strain of* P. ostreatus* (Pl 22 Em), available at the culture collection of the Bioprocess Engineering and Biotechnology Department (Federal University of Paraná, Curitiba, Brazil) was reactivated in PDA dishes and after 7 days of growth, 4 disks of 7 mm diameter were transferred to Czapek liquid medium containing the antibiotic cephalexin (0.08 g/L). After 5 days of growth at 30°C and 120 rpm, the mycelium was separated from the residual medium by a sieve, homogenized with a spatula and resuspended in the residual medium to a lower final volume (10% of the initial volume). 0.2 mL of the homogenized mycelium (containing 4% of dry biomass) was transferred to the fermentation flasks, which were manually homogenized and incubated for 5 days (optimization studies) or for 3 to 7 days (kinetic study).

### 2.3. Experimental Designs

Fermentations were prepared as described in Section 2.2. Tables 1 and 2 present the chosen variables and levels for the Plackett-Burman Design and Central Composite Design experiments, respectively.

Analysis of the results and determination of the mathematical model were performed using the software Statistica 5.0 (Statsoft, USA), and determination of the optimal levels was performed through the Solver Excel tool (Microsoft, USA).

### 2.4. Extraction of the Enzymes

Enzymes produced by solid-state fermentation were extracted by solid-liquid extraction using sodium phosphate buffer as solvent (NaH_2_PO_4_·H_2_O, 50 mM, pH 7.0) [21]. The fermented material was manually homogenized and weighed (around 1 g) and the extraction buffer was added in the proportion of 1 : 10 (w/w). A protease inhibitor (phenylmethylsulfonyl fluoride, PMSF 1 mM) was added to the extraction mixture. The mixture was homogenized in vortex for 1 min and centrifuged for 7,500 g, 4°C, 45 min. The supernatant was separated and submitted to analyses.

### 2.5. Laccase Activity Assay

The enzymatic activity of laccase was assayed by the oxidation of ABTS (2,2′-azino-bis-3-ethylbenzthiazoline-6-sulphonic acid). The reaction mixture contained 100 *μ*L of ABTS 20 mM (in sodium citrate buffer 0.1 M, pH 3.0), sample (usually 20–50 *μ*L), and sodium citrate buffer (C_6_H_8_O_7_·H_2_O 0.1 M, pH 3.0) up to 1 mL. Oxidation of ABTS was followed by absorbance increase at 420 nm (*ε* = 36,000 M^−1^ cm^−1^). The enzyme activity was expressed in international units (U), where one unit of enzyme activity is defined as the amount of enzyme that oxidizes 1 *μ*mol of substrate in 1 min.

### 2.6. Biological Delignification

Erlenmeyer flasks containing 3 g of sugarcane bagasse (particle size between 0.8 and 2 mm 50% and <0.8 mm 50%) were autoclaved and humidified with a saline solution (sterilized by filtration, 10 mL/g bagasse) presenting the following constant composition: MgSO_4_·7H_2_O (0.3 g/L), FeSO_4_·7H_2_O (0.005 g/L), MnSO_4_·H_2_O (0.00156 g/L), ZnSO_4_·7H_2_O (0.0014 g/L), CaCl_2_ (0.3 g/L), CoCl_2_ (0.002 g/L), KH_2_PO_4_ (1.5 g/L), and pH 5.5. Yeast extract (sterilized by autoclaving at 121°C, 1 atm, 15 min) and copper sulfate (sterilized by filtration) were added to the saline solution to reach final concentrations of 6.4 g/L and 173 *μ*M, respectively. Ferulic acid (sterilized by filtration) was added after 48 h of fermentation to a final concentration of 1.86 mM. The inoculum was prepared according to Section 2.2. and the incubation was performed at 29°C. Physicochemical analyses of the biotreated bagasse were performed as described in Section 2.1.

## 3. Results and Discussion

### 3.1. Screening of Significant Variables Affecting Laccase Production: Plackett-Burman Design

Table 3 presents the results of the Plackett-Burman experiments to select significant variables to be optimized for laccase production by the strain* P. ostreatus* Pl 22 Em.

According to the Pareto analysis, the variables that presented significant effects on laccase production at the confidence level of 90% were copper sulfate and ammonium sulfate concentrations, both presenting positive standardized effects of 11.25 and 5.003, respectively (absolute values, *R* ^2^ = 0.96873).

As far as the effect of copper sulfate is concerned, the addition of copper as an inducer of laccase production has been already reported in literature. Different studies have shown that laccase production is regulated by metal ions such as Cu^2+^ and Fe^3+^ by gene expression induction or through translational or posttranslational regulation [22, 23]. Palmieri et al. [4] demonstrated that the addition of copper sulfate 150 *μ*M to a* P. ostreatus* (Jacq.:Fr.) Kummer (type Florida) liquid culture medium caused a 30-fold increase in total laccase activity, and Hou et al. [5] reported a 4.5-fold increase in laccase activity in* P. ostreatus* (strain 32, Dalian Institute of Mushroom Study) when Cu^2+^ 1 mM was added to the liquid culture medium. Baldrian and Gabriel [24] concluded that Cu^2+^ not only induces laccase by the expression of laccase genes in* P. ostreatus *CCBAS-447 (Institute of Microbiology, Academy of Sciences of the Czech Republic) but also positively affects activity and stability of the enzyme.

As far as the effect of ammonium sulfate is concerned, the production of ligninolytic enzymes has been associated with the secondary metabolism and with conditions of limited nitrogen for many white rot fungi, including the model organism for laccase production and lignin degradation* Phanerochaete chrysosporium* [25]. For* P. ostreatus *(HAI 493, Nextlab, Hawaii), however, a higher concentration of nitrogen in the medium did not repress but rather slightly stimulated mineralization of lignin, as reported by Stajić et al. [26]. Kaal et al. [25] also suggested that several white rot fungi strains, including* P. ostreatus*, produce higher ligninolytic enzyme activities in response to a nitrogen-rich medium.

### 3.2. Comparison between Organic and Inorganic Nitrogen Sources and Evaluation of Ferulic Acid as Inducer of Laccase Activity Production

The comparison between the laccase activities produced by the strain* P. ostreatus* Pl 22 Em when different sources of nitrogen were used and when ferulic acid was added is presented in Table 4. Using yeast extract (7.5 g/L, containing 7% total nitrogen) instead of ammonium sulfate (2.5 g/L, containing 21% nitrogen) for the same concentration of total nitrogen caused an increase of 5.7-fold in laccase production (44.23 ± 2.44 versus 9.942 ± 1.97 U/g dry substrate after 5 days of fermentation).

These results are in accordance with Hou et al. [5], who demonstrated that the most suitable nitrogen sources for laccase production by* P. ostreatus* (strain 32) were peptone and yeast extract, in comparison with urea, ammonium sulfate, and ammonium tartrate. These organic nitrogen sources increased laccase activity in 1.55, 1.99, and 1.46, respectively, (peptone) and 1.40, 1.79, and 1.32, respectively (yeast extract). Mishra and Kumar [27] also demonstrated that, regarding enhancement of laccase production in solid-state fermentation by* P. ostreatus *MTCC1804 (Institute of Microbial Technology, Chandigarh, India), yeast extract was preferred to inorganic nitrogen sources, reaching 23 U/g dry substrate against 2.2 U/g (without nitrogen supplementation), 10.11 U/g (with ammonium sulfate), and 13.0 U/g (with urea). Highest activity, however, was obtained in the presence of cyanobacterial biomass and copper sulfate 1 mM (65 U/g after 10 days). These results may be attributed to the presence of some additives (nutrients/activators) and favorable C : N ratio of the organic nitrogen sources [27].

Addition of ferulic acid to the copper containing medium further enhanced laccase production (2.0-fold) by the strain* P. ostreatus* Pl 22 Em (89.18 ± 3.95 versus 44.23 ± 2.44 U/g dry substrate). Ferulic acid is a known inducer of laccase production. The structure of this organic acid is similar to that of coniferyl alcohol, the most abundant monolignol of the three lignin precursors [28]. Vanhulle et al. [29] reported a positive effect of ferulic acid 0.5 mM on laccase production by* P. ostreatus* IT01 in submerged fermentation with glucose and lactose as substrates. A peak of laccase activity (around 7,500 U/L) was observed at the 15th day of fermentation (3-fold increase when compared to control). Ferulic acid was also shown to be the best inducer of laccase activity in* Pleurotus sajor-caju* [28]. In solid-state fermentation, Meza et al. [30] reported a laccase activity of approximately 70 U/g of sugarcane bagasse in the presence of ferulic acid 10 mM, produced by* Pycnoporus cinnabarinus* after 10 days, in contrast with around 10 U/g without inducers. It is worth noting that the values achieved in this study in the presence of copper sulfate (44.23 ± 2.44 U/g) and ferulic acid (89.18 ± 3.95 U/g) after 5 days of fermentation were promising when compared to other results reported in literature.

### 3.3. Determination of the Mathematical Model of Laccase Production through the Response Surface Methodology: Central Composite Design and Kinetics of Laccase Production under Optimized Conditions

Table 5 presents the results of 16 experiments to evaluate the effect of yeast extract, copper sulfate, and ferulic acid concentrations on laccase production by solid-state fermentation in sugarcane bagasse.

According to Table 6, the linear and quadratic terms of all three variables significantly affected the response. Interaction effects (not shown) were not significant. The mathematical model of laccase production can be given by the following equation:

(1) Laccase  activity(U/g)=−249.9+41.53[YE] −3.236YE2+2.071[Cu2+] −0.0060Cu2+2+106.7[Fer] −28.68Fer2,

where concentrations of yeast extract (YE), copper (Cu^2+^), and ferulic acid (Fer) are given in g/L, *μ*M, and mM, respectively.

The predicted model indicated that the maximum laccase activity (161.3 U/g dry substrate) would be obtained at the following conditions: yeast extract 6.417 g/L, Cu^2+^ 172.6 *μ*M, and ferulic acid 1.860 mM.

Experiments for the verification of the predicted model (Table 7) revealed a correlation coefficient (*R* ^2^) of 0.8963, the most significant differences being obtained at the lowest and highest levels (24% and 44%, resp.). However, when these points were not considered, the model described the laccase production as a function of yeast extract, CuSO_4,_ and ferulic acid concentrations, within the range of 5.5 to 8.5 g/L, 112.5 to 187.5 *μ*M, and 1.5 to 2.5 mM, respectively, with a correlation coefficient of 0.9798.

Other values reported in the literature for laccase production by* P. ostreatus* in solid-state fermentation are 65.42 U/g with copper as inducer [27], 9 U/g without inducers [31], and 36 U/g without inducers [21].

**Figure 1:**
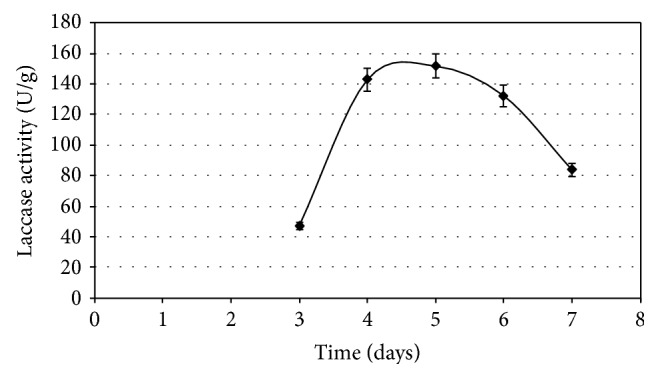
Kinetics of laccase production on solid-state fermentation of sugarcane bagasse by* P. ostreatus* 22 Em under optimized conditions: 6.4 g/L yeast extract, 172.6 *μ*M CuSO_4_, and 1.86 mM ferulic acid.

Figure 1 presents the kinetics of laccase production under optimized conditions. The peak of laccase activity (151.6 U/g) was obtained between the 4th day and the 5th day of solid-state fermentation. Other values of laccase productivity reported in the literature are 80 U/mL after 12 days in liquid culture of* P. ostreatus* hybrids without exogenous inducers [6], 12.2 U/mL after 18 days of liquid fermentation by* P. ostreatus* [32], 90 U/g of sugarcane bagasse produced by* Pycnoporus cinnabarinus* after 14 days of solid-state fermentation in columns and activities near 80 U/g after 6 days [33], and 70 U/g of sugarcane bagasse, produced by* Pycnoporus cinnabarinus* after 10 days [30]. The maximum value of laccase production achieved with the strain* P. ostreatus* Pl 22 Em in this study (151.6 U/g) after 5 days of solid-state fermentation was promising in terms of enzyme activity and productivity.

Further enhancement in the production of laccase could be achieved by other strategies such as coculturing ligninolytic fungi and filamentous microfungi, as demonstrated by Cupul et al. [34]. These authors reported an increase in laccase activity from 4,881.0 to 12,382.5 U/mg protein, when the fungus* Paecilomyces carneus* was inoculated to a culture of the ligninolytic fungus* Trametes maxima*. Also, studies on gene expression could be performed since the physiological behavior of many laccase producing organisms suggests the presence of elements responsive to metals (MRE), xenobiotics (XRE), heat shock (HSE), and oxidative stress (ARE, antioxidant response element) within the promoter region of laccase genes [35]. Regarding heterologous expression, advantages are more related to providing favorable conditions for genetic studies and enzyme characterization than to the production of high activities. Macellaro et al. [36] developed a process for heterologous production of the high redox potential* Pleurotus ostreatus* laccase POXA1b and one of its variants, 1H6C, using* Aspergillus niger* as a host, and obtained production levels of 35,000 U/L and 60,000 U/L, respectively. You et al. [37] reached a maximum laccase activity of 685.8 U/L through heterologous expression of a* Ganoderma lucidum* laccase in* Pichia pastoris*.

### 3.4. Characterization of the Sugarcane Bagasse and Kinetics of Biological Delignification

The culture conditions optimized for laccase production were applied in the biotreatment of sugarcane bagasse with the aim of evaluating lignin degradation. The sugarcane bagasse received from the industry presented the following particle size distribution: 21.2% between 0.8 and 2.0 mm, 22.8% < 0.8 mm, and 56% > 2.0 mm. After grinding and classification, the particle size distribution changed to 42.4% between 0.8 and 2.0 mm and 57.6% < 0.8 mm. The physicochemical composition of sugarcane bagasse before and after the biotreatment of solid-state fermentation is presented in Table 8. All values of lignin percentage were statistically different among samples. Extractives in sugarcane bagasse can be represented by waxes, pigments, alkaloids, terpenes, flavonoids, coumarins, tannins, sugars, and saponins [38]. Reduction of lignin content was of 5.53% after 5 days and of 11.1% after 15 days of solid-state fermentation. Of these percentage reductions, 3.41% were not due to fungal degradation but to the addition of nutrients for optimized laccase production.

The process developed by Pellinen et al. [39] to delignify kraft pulp and chemithermomechanical pulp (CTMP) using* Phanerochaete chrysosporium* presented delignification times of around two weeks, the kappa number (residual lignin) being reduced from 33 to less than 10 for the kraft pulp, and the lignin content decreasing from 26.5% to 21.3% for the CTMP. Delignification of sugarcane bagasse by* Ceriporiopsis subvermispora* during 30 days resulted in a pulp yield of 46–54% [40]. Meza et al. [41] presented a process for biological delignification of sugarcane bagasse and simultaneous production of laccases that yielded a laccase activity of 80 U/g and an energy economy of 50% during pulping and refining, after 28 days of fungal treatment. Knežević et al. [42] reported a reduction of 34.1% in lignin content of wheat straw after 14 days of cultivation of* Dichomitus squalens*.

The process of delignification is very complex and involves the synergistic action of laccases and peroxidases. Although laccases are directly involved in delignification through the oxidation of phenolic structures, their production level is not necessarily related to the rate of delignification. Also, lignin degradation can occur until the latest stages of fermentation, even if the peak of enzymatic activity is achieved earlier [42]. The fungus* P. ostreatus* 22 Em, which demonstrated to be a high producer of laccases on sugarcane bagasse after 5 days of solid-state fermentation, presented a moderate efficiency of delignification with the highest rate achieved between the 5th day and the 10th day. However, delignification is not the only beneficial action of laccases with respect to the use of lignocellulosic biomass. Although this action facilitates the cellulose hydrolysis and glucose release, there is also a contribution related to the degradation of phenolic compounds released during pretreatment, which reduces the toxicity of the broth for a subsequent fermentation [43, 44].

## 4. Conclusions

The level of laccase activity produced by* P. ostreatus* Pl 22 Em in solid-state fermentation of sugarcane bagasse was significantly affected by the concentrations of nitrogen source, copper sulfate, and ferulic acid. The use of an organic nitrogen source (yeast extract) provided and increase of 5.7-fold in laccase production, in comparison with the inorganic source (ammonium sulfate). The predicted model indicated that the maximum laccase activity (161.3 U/g of sugarcane bagasse) would be obtained at the following conditions: yeast extract 6.417 g/L, Cu^2+^ 172.6 *μ*M, and ferulic acid 1.860 mM. Experimentally, the maximum laccase activity of 151.6 U/g was produced, under optimized conditions, at the 5th day of solid-state fermentation, which is higher than that obtained in other solid-state fermentations so far reported on sugarcane bagasse. On the other hand, the process of biological delignification reduced the lignin content of sugarcane bagasse from 31.89% to 26.36% after 5 days and to 20.79% after 15 days of solid-state fermentation, which represents a moderate efficiency of delignification. The highest rate of lignin degradation was achieved between the 5th day and the 10th day.

## Figures and Tables

**Table 1:** Plackett-Burman design to select significant variables to be optimized in the production of laccase by solid state fermentation in sugarcane bagasse.

Levels	Variables							
	pH	*A*_*w*_^a^	*T*	CuSO_4_	(NH_4_)_2_SO_4_	KH_2_PO_4_	Asn^b^	YE^c^
			°C	*μ*M	g/L	g/L	g/L	g/L
−1	5.0	0.993	25	0	1.5	1	0	0
+1	6.0	0.999	33	150	2.5	2	0.6	0.5

^a^Water activity; ^b^asparagine; ^c^yeast extract.

**Table 2:** Central composite design for the modeling of laccase production by solid-state fermentation in sugarcane bagasse.

Levels	Variables		
	Yeast extract g/L	CuSO_4_ *μ*M	Ferulic acid mM
−1.68	1.96	24	0.32
−1	4	75	1
0	7	150	2
+1	10	225	3
+1.68	12.04	276	3.68

**Table 3:** Results of laccase activity obtained for the Plackett-Burman experiments after 5 days of solid-state fermentation on sugarcane bagasse.

	Variables and corresponding levels								Activity
	pH	*A*_*w*_^a^	*T* ^b^	[Cu^2+^]^c^	[N]^d^	[PK]^e^	[Asn]^f^	[YE]^g^	U/g
1	1	−1	1	−1	−1	−1	1	1	2.091
2	1	1	−1	1	−1	−1	−1	1	7.420
3	−1	1	1	−1	1	−1	−1	−1	3.278
4	1	−1	1	1	−1	1	−1	−1	7.366
5	1	1	−1	1	1	−1	1	−1	11.45
6	1	1	1	−1	1	1	−1	1	4.294
7	−1	1	1	1	−1	1	1	−1	6.825
8	−1	−1	1	1	1	−1	1	1	10.94
9	−1	−1	−1	1	1	1	−1	1	11.18
10	1	−1	−1	−1	1	1	1	−1	3.729
11	−1	1	−1	−1	−1	1	1	1	1.914
12	−1	−1	−1	−1	−1	−1	−1	−1	2.755
C^h^	0	0	0	0	0	0	0	0	7.318
C′	0	0	0	0	0	0	0	0	6.727

Note: enzyme activities in units per gram of dry substrate.

^a^Water activity; ^b^temperature; ^c^CuSO_4_ concentration; ^d^(NH_4_)_2_SO_4_ concentration; ^e^KH_2_PO_4_ concentration; ^f^asparagine concentration; ^g^yeast extract concentration; ^h^C and C′ represent the duplicates of the intermediate level.

**Table 4:** Effect of inorganic and organic nitrogen sources and different inducers, Cu^2+^ and ferulic acid (Fer), on the level of laccase activity produced by the strain *P.  ostreatus* 22 Em after 5 days of fermentation on sugarcane bagasse.

N source (g/L)	Inducer	U/g dry substrate
Ammonium sulfate		
2.5	CuSO_4_ 150 *μ*M	9.942 ± 1.97
Yeast extract		
2.5	0	2.970 ± 0.651
2.5	CuSO_4_ 150 *μ*M	44.23 ± 2.44
2.5	CuSO_4_ 150 *μ*M + Fer 2 mM	89.18 ± 3.95
7.5	CuSO_4_ 150 *μ*M	56.25 ± 5.08

**Table 5:** Results of laccase activity obtained for the central composite design experiments after 5 days of solid-state fermentation on sugarcane bagasse.

	Variables			Response
	Yeast extract g/L	CuSO_4_ *μ*M	Ferulic acid mM	Activity U/g
1	4	75	1	61.30
2	10	75	1	51.02
3	4	225	1	98.12
4	10	225	1	58.80
5	4	75	3	50.90
6	10	75	3	46.60
7	4	225	3	68.80
8	10	225	3	65.60
9	1.96	150	2	82.19
10	12.04	150	2	52.75
11	7	24	2	62.33
12	7	276	2	35.91
13	7	150	0.32	48.03
14	7	150	3.68	89.43
15	7	150	2	158.8
16	7	150	2	149.4

Note: enzyme activities in units per gram of dry substrate.

**Table 6:** Regression coefficients and identification of significant variables (*P* < 0.05) for laccase production using central composite design, *R* ^2^ = 0.8753.

Factor	Coefficients	Standard error	*t*-value	*P* value
Intercept	−249.9	80.11	−3.120	0.02059
Yeast extract (L^a^)	41.53	12.19	3.405	0.01440
Yeast extract (Q^b^)	−3.236	0.7220	−4.482	0.004182
Cu²^+^ (L)	2.071	0.4551	4.550	0.003890
Cu²^+^ (Q)	−0.0060	0.00116	−5.483	0.001539
Ferulic acid (L)	106.7	34.13	3.127	0.02040
Ferulic acid (Q)	−28.68	6.498	−4.414	0.004501

^a^Linear; ^b^quadratic.

**Table 7:** Experiments for the verification of the predicted model of laccase production after 5 days of solid-state fermentation on sugarcane bagasse.

Yeast extract (g/L)	CuSO_4_ (*μ*M)	Ferulic acid (mM)	Predicted (U/g)	Experimental (U/g)
4.0	75	1.0	64.04	79.74 ± 7.94
5.5	112.5	1.5	133.2	114.7 ± 7.17
6.4	172.6	1.86	161.3	155.3 ± 5.92
7.0	150	2.0	156.6	149.2 ± 4.95
8.5	187.5	2.5	134.2	122.9 ± 5.12
10	225	3.0	66.01	95.17 ± 7.75

Note: enzyme activities in units per gram of dry substrate.

**Table 8:** Physicochemical composition of sugarcane bagasse, before and after biotreatment.

	Sugarcane bagasse	Bagasse prepared for biotreatment	Biotreated bagasse 5 days	Biotreated bagasse 10 days	Biotreated bagasse 15 days
Lignin (%)	31.89	28.48	26.36	22.37	20.79
Holocellulose (%)	63.36	63.30	64.38	67.84	69.12
Extractives (%)	2.15	5.88	5.57	6.05	6.17
Ashes (%)	2.60	2.34	3.69	3.74	3.92
Moisture (%)	7.57	91.6	88.28	87.71	85.91

Note: percentages of lignin, holocellulose, extractives, and ashes are in moisture free basis. Average standard deviations were 0.367 for lignin, 0.165 for extractives, 0.233 for ashes, and 0.0587 for moisture.
